# Accuracy of breast MRI in evaluating nodal status after neoadjuvant therapy in invasive lobular carcinoma

**DOI:** 10.1038/s41523-021-00233-9

**Published:** 2021-03-05

**Authors:** Mary Kathryn Abel, Heather Greenwood, Tatiana Kelil, Ruby Guo, Case Brabham, Nola Hylton, Jasmine Wong, Michael Alvarado, Cheryl Ewing, Laura J. Esserman, Judy C. Boughey, Rita A. Mukhtar

**Affiliations:** 1 University of California, San Francisco School of Medicine, San Francisco, CA USA; 2 Department of Surgery, University of California, San Francisco, San Francisco, CA USA; 3 Department of Radiology and Biomedical Imaging, University of California, San Francisco, San Francisco, CA USA; 4 Department of Surgery, Mayo Clinic, Rochester, MN USA

**Keywords:** Breast cancer, Cancer imaging

## Abstract

Neoadjuvant therapy in breast cancer can downstage axillary lymph nodes and reduce extent of axillary surgery. As such, accurate determination of nodal status after neoadjuvant therapy and before surgery impacts surgical management. There are scarce data on the diagnostic accuracy of breast magnetic resonance imaging (MRI) for nodal evaluation after neoadjuvant therapy in patients with invasive lobular carcinoma (ILC), a diffusely growing tumor type. We retrospectively analyzed patients with stage 1–3 ILC who underwent pre-operative breast MRI after either neoadjuvant chemotherapy or endocrine therapy at our institution between 2006 and 2019. Two breast radiologists reviewed MRIs and evaluated axillary nodes for suspicious features. All patients underwent either sentinel node biopsy or axillary dissection. We evaluated sensitivity, specificity, negative and positive predictive values, and overall accuracy of the post-treatment breast MRI in predicting pathologic nodal status. Of 79 patients, 58.2% received neoadjuvant chemotherapy and 41.8% neoadjuvant endocrine therapy. The sensitivity and negative predictive value of MRI were significantly higher in the neoadjuvant endocrine therapy cohort than in the neoadjuvant chemotherapy cohort (66.7 vs. 37.9%, *p* = 0.012 and 70.6 vs. 40%, *p* = 0.007, respectively), while overall accuracy was similar. Upstaging from clinically node negative to pathologically node positive occurred in 28.0 and 41.7%, respectively. In clinically node positive patients, those with an abnormal post-treatment MRI had a significantly higher proportion of patients with ≥4 positive nodes on pathology compared to those with a normal MRI (61.1 versus 16.7%, *p* = 0.034). Overall, accuracy of breast MRI for predicting nodal status after neoadjuvant therapy in ILC was low in both chemotherapy and endocrine therapy cohorts. However, post-treatment breast MRI may help identify patients with a high burden of nodal disease (≥4 positive nodes), which could impact pre-operative systemic therapy decisions. Further studies are needed to assess other imaging modalities to evaluate for nodal disease following neoadjuvant therapy and to improve clinical staging in patients with ILC.

## Introduction

One advantage of neoadjuvant therapy in the treatment of breast cancer is the potential to downstage the axilla and reduce the extent of morbid axillary surgery^1–6^. The nodal response to neoadjuvant therapy can impact both surgical planning and the appropriate duration and type of neoadjuvant therapy. Additionally, identifying patients with ≥4 involved nodes preoperatively can impact systemic therapy choices^7^. As such, developing ways to accurately evaluate the axilla with imaging is important in the management of breast cancer patients. Given its prevalence in evaluating the in-breast response to neoadjuvant therapy, there is increasing interest in the ability of breast magnetic resonance imaging (MRI) to determine axillary status as well.

Studies have evaluated the diagnostic accuracy of breast MRI for predicting axillary lymph node status after neoadjuvant chemotherapy, with accuracy ranging from 60 to 87%^8–13^. However, there are currently limited data on the accuracy of breast MRI for predicting nodal status after neoadjuvant endocrine therapy, and no data specifically evaluating accuracy for patients with invasive lobular carcinoma (ILC) of the breast. ILC is the second most common type of breast cancer and grows in a diffuse pattern due to the lack of adhesion protein E-cadherin^14^. Patients with ILC and a positive sentinel lymph node have a higher burden of additional nodal disease than the more common invasive ductal carcinoma (IDC)^15^. Standard breast imaging tools like mammography are known to have a lower sensitivity for ILC than IDC^16–18^.

For these reasons, it is important to establish the accuracy of breast MRI in the evaluation of the axilla in this unique breast cancer subtype. In this study, we evaluated a cohort of ILC patients treated with either neoadjuvant chemotherapy or endocrine therapy and determined the accuracy of post-treatment MRI for predicting pathologic nodal status in both clinically node negative and clinically node positive patients.

## Results

### Patient population

Of 487 patients treated for stage I–III ILC at our institution between 2006 and 2019, 119 (24.4%) received neoadjuvant chemotherapy or neoadjuvant endocrine therapy. Of these, 84 patients (70.5%) received post-treatment breast MRI prior to surgery. Five patients were excluded from analysis due to missing pathology data, leaving a total of 79 patients who met the inclusion criteria for this study. Of these patients, 46 (58.2%) were treated with neoadjuvant chemotherapy (Adriamycin-based in 58.7%, on clinical trial in 19.6%, non-Adriamycin based in 10.9%, and unknown type in 10.9%). The remaining 33 (41.8%) individuals were treated with neoadjuvant endocrine therapy, with most receiving an aromatase inhibitor (*n* = 25, 75.8%) for a minimum of three months prior to surgery (median: 208 days, range: 95–362 days). An additional 4 patients received tamoxifen (12.1%, median length: 163 days, range 123–198 days), and 4 patients had unknown endocrine therapy type (12.1%).

The demographic and clinicopathologic data are described in Table 1. The mean age was 56.5 years (range: 38.6–83.6 years). Most patients had grade 2 tumors (65.8%) and estrogen-receptor positive (ER+), progesterone receptor-positive (PR+), and human epidermal growth factor receptor 2-negative (HER2-) disease (52.0%). Seventy-one (89.9%) patients in this cohort had a breast MRI prior to starting neoadjuvant therapy in addition to the post-treatment breast MRI. Additionally, 40 (50.6%) patients received a pre-treatment axillary ultrasound as part of their clinical evaluation. ILC subtype was classic in 60 (76.0%) patients, while 11 (13.9%) patients had pleomorphic ILC and 8 (10.1%) had mixed ILC/IDC. Thirty patients (38.0%) were clinically node positive based on pre-treatment fine needle aspiration (FNA) or core biopsy, which was prompted by abnormal physical examination and imaging findings in 19 (63.3%) patients and by abnormal imaging alone in 10 (33.3%) patients.

**Table 1:** Patient and tumor characteristics.

Variable	Overall (*n* = 79)	Chemotherapy (*n* = 46)	Endocrine therapy (*n* = 33)	*P*-Value
Age at diagnosis (years, SD)	56.5 (9.7)	54.1 (8.3)	59.9 (10.5)	0.159
Receptor subtype (*n* = 75)				0.022
ER+/PR+/HER2−	39 (52.0)	21 (46.7)	18 (60.0)	
ER+/PR−/HER2−	24 (32.0)	12 (26.7)	12 (40.0)	
Triple negative	1 (1.3)	1 (2.2)	0 (0.0)	
HER2+	11 (14.7)	11 (24.4)	0 (0.0)	
ILC Grade				0.140
1	23 (29.1)	11 (23.9)	12 (36.4)	
2	52 (65.8)	31 (67.4)	21 (63.6)	
3	4 (5.1)	4 (8.7)	0 (0.0)	
ILC Stage				0.001
1	33 (41.8)	13 (28.3)	20 (60.6)	
2	31 (39.2)	26 (56.5)	5 (15.2)	
3	15 (19.0)	7 (15.2)	8 (24.2)	
Lymphovascular Invasion	7 (8.9)	5 (10.9)	2 (6.1)	0.458
Radiation (*n* = 77)	52 (67.5)	32 (69.6)	20 (64.5)	0.643
Treatment type (*n* = 77)				0.070
Lumpectomy without radiation	7 (9.1)	3 (6.5)	4 (12.9)	
Lumpectomy with radiation	25 (32.5)	11 (23.9)	14 (45.2)	
Mastectomy	18 (23.4)	11 (23.9)	7 (22.6)	
Mastectomy with radiation	27 (35.1)	21 (45.7)	6 (19.4)	
Axillary surgery				0.023
Sentinel lymph node biopsy	36 (45.6)	16 (34.8)	20 (60.6)	
Axillary dissection	43 (54.4)	30 (65.2)	13 (39.4)	
Clinical node status				<0.001
Positive	30 (38.0)	22 (47.8)	8 (24.2)	
Negative	49 (62.0)	24 (52.2)	25 (75.8)	
Pathologically node positive at surgery	44 (55.7)	29 (63.0)	15 (45.5)	0.067
1–3 Positive nodes	25 (31.7)	20 (43.5)	5 (15.2)	
4–9 Positive nodes	11 (13.9)	5 (10.9)	6 (18.2)	
10 or more positive nodes	8 (10.1)	4 (8.7)	4 (12.1)	
Size of cancer in positive nodes, cm (mean, SD) (*n* = 44)	0.87 (0.67)	0.79 (0.56)	1.02 (0.84)	0.083
Patients with positive nodes ≥ cm (*n* = 44)	16 (36.4)	11 (37.9)	5 (33.3)	0.764

Compared to the neoadjuvant endocrine therapy cohort, patients who received neoadjuvant chemotherapy were more likely to present with stage 2 or 3 disease (71.7 vs. 39.4%, *p* = 0.001) and be clinically node positive prior to initiating neoadjuvant therapy (47.8 vs. 24.2%, *p* < 0.001, Table 1). There was one patient with triple-negative disease and 11 (24.4%) patients with HER2 positive disease in the neoadjuvant chemotherapy cohort, and none in the neoadjuvant endocrine therapy cohort. There were no statistically significant differences in age, grade, presence of lymphovascular invasion, the number of positive nodes at surgery, type of surgical treatment, or size of axillary tumor at pathology between those treated with neoadjuvant endocrine therapy and neoadjuvant chemotherapy.

### MRI and pathology findings

**Fig. 1:**
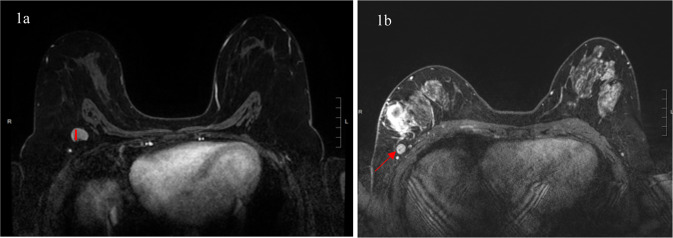
Abnormal lymph node findings on breast MRI. **a**, **b** Examples of lymph node abnormalities on breast MRI. **a** Left: shows increased cortical thickening (≥3 mm) of an axillary lymph node in the right axilla (red line). **b** Right: shows an example of an axillary lymph node with rounded morphology in the right axilla and asymmetry when compared to the contralateral axilla (red arrow).

Overall, a total of 32 patients (40.5%) had at least one abnormal feature involving axillary nodes on post-treatment MRI (Fig. 1a, b). Of these patients, 21 (65.6%) had at least one positive lymph node on surgical pathology. There were 47 patients with normal appearing nodes on post-treatment MRI, of whom 23 (48.9%) had at least one positive node at surgery.

Forty-four (55.7%) patients had at least one pathologically positive node at surgical excision, of which 38 (86.4%) were macrometastases (15 with extracapsular extension) and 6 (13.6%) were micrometastases. A total of 6 patients (13.6%) had evidence of treatment effect in lymph nodes. Each abnormal imaging feature was significantly associated with larger size of tumor deposit. For those with cortical thickening, rounded morphology, loss of fatty hilum, or asymmetry versus those without, mean tumor deposit size was 1.3 cm vs. 0.6 cm, 1.3 cm vs. 0.7 cm, 1.3 cm vs. 0.7 cm, and 1.2 cm vs. 0.5 cm, respectively (all *p* < 0.006). There was no association between histologic variant of ILC and pathologic nodal status, size of tumor deposit in nodes, or presence of treatment effect.

### Neoadjuvant chemotherapy cohort

**Fig. 2:**
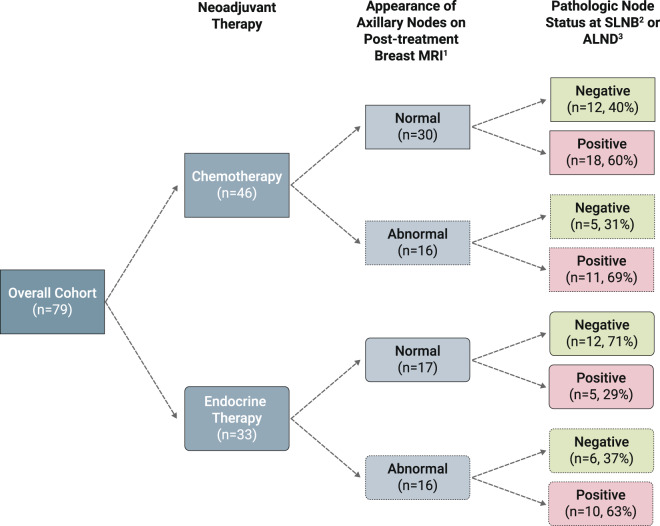
Pathologic node status by type of neoadjuvant therapy and MRI appearance of axillary nodes. Appearance of axillary nodes on MRI and subsequent pathologic node status following neoadjuvant chemotherapy or neoadjuvant endocrine therapy. ^1^Appearance of axillary nodes was determined based on nodal BIRADS features. ^2^SLNB: sentinel lymph node biopsy. ^3^ALND: axillary lymph node dissection.

Of the 46 patients treated with neoadjuvant chemotherapy, 16 (34.8%) had abnormal nodes identified on post-treatment breast MRI, with increased cortical thickness being the most common abnormal axillary imaging feature (Fig. 2, Table 2). Of these 16 patients, 11 (68.8%) had nodes containing metastatic disease at the time of surgery. The remaining 30 patients had normal appearing nodes on breast MRI; of these patients, 18 (60.0%) had pathologically positive nodes. The sensitivity of abnormal nodal features on breast MRI in predicting nodal status at surgery was 37.9%, while the specificity was 70.6% (Table 3). The positive and negative predictive values were 68.8 and 40.0%, respectively, with an overall accuracy of 50%.

**Table 2:** Appearance of axillary nodes on post-treatment breast MRI.

Axillary imaging findings	Neoadjuvant chemotherapy (*n* = 46)	Neoadjuvant endocrine therapy (*n* = 33)
BIRADS Features		
Cortical thickness (≥3 mm)	14 (30.4)	12 (40.0)
Rounded morphology	7 (15.2)	7 (21.2)
Loss of fatty hilum	7 (15.2)	7 (21.2)
Asymmetry^a^	13 (28.3)	16 (48.5)
Any abnormal BIRADS feature	16 (34.8)	16 (48.5)
Number of abnormal nodes		
None	30 (65.2)	17 (51.5)
Single	8 (17.4)	5 (15.2)
Multiple	7 (15.2)	9 (27.3)
Missing	1 (2.2)	2 (6.1)
Axillary level of involvement		
None	30 (65.2)	17 (51.5)
Level I	10 (21.7)	11 (33.3)
Level I and II	5 (10.9)	0 (0.0)
Level I, II, and III	0 (0.0)	3 (9.1)
Missing	1 (2.2)	2 (6.1)

^a^Unable to evaluate for 4 patients in chemotherapy cohort and 2 in endocrine therapy cohort.

**Table 3:** Statistical tests evaluating the predictive value of MRI in detecting nodal status after neoadjuvant therapy overall and by clinical node status.

Overall Cohort			
	**Neoadjuvant chemotherapy (*n*** = 46)	**Neoadjuvant endocrine therapy (*n*** = 33)	***P*-Value**
Sensitivity	37.9%	66.7%	0.0116
Specificity	70.6%	66.7%	0.7117
Negative predictive value	40.0%	70.6%	0.0072
Positive predictive value	68.8%	62.5%	0.5594
Overall accuracy	50.0%	66.7%	0.1393
Neoadjuvant chemotherapy Cohort			
	**Clinically node negative (*n*** = 24)	**Clinically node positive (*n*** = 22)	***P*-Value**
Sensitivity	20.0%	47.4%	0.0485
Specificity	78.6%	33.3%	0.0019
Negative predictive value	57.9%	9.1%	0.0005
Positive predictive value	40.0%	81.2%	0.0044
Overall accuracy	54.2%	45.5%	0.5555
Neoadjuvant endocrine therapy cohort			
	**Clinically node negative (*n*** = 25)	**Clinically node positive (*n*** = 8)	***P*-Value**
Sensitivity	42.9%	87.5%	0.0277
Specificity	66.7%	N/A^1^	
Negative predictive value	75.0%	0.0%	0.0002
Positive predictive value	33.3%	100.0%	0.001
Overall accuracy	60.0%	N/A^a^	

^a^There were no clinically node positive patients who received endocrine therapy and were pathologically node negative; as such, specificity and accuracy of post-treatment MRI cannot be calculated for this subset.

Sensitivity and PPV were significantly higher in clinically node positive patients compared to clinically node negative patients (47.4 vs. 20%, *p* = 0.0485 and 81.2 vs. 40%, *p* = 0.0044, Table 3). Specificity and NPV were significantly higher in clinically node negative patients compared to clinically node positive patients (78.6 vs. 33.3%, *p* = 0.0019 and 57.9 vs. 9.1%, *p* = 0.0005, Table 3). Overall accuracy was similar between the clinically node positive and clinically node negative groups (54.2 and 45.5%, respectively).

### Neoadjuvant endocrine therapy cohort

For the neoadjuvant endocrine therapy cohort, 16 patients (48.5%) had abnormal nodes by suspicious features identified on post-treatment breast MRI (Fig. 2). The most common abnormal axillary imaging feature was asymmetry compared to the contralateral side (Table 2). Of the 16 patients with abnormal nodal imaging, 10 (62.5%) had positive nodes on pathology. Seventeen patients had normal appearing nodes on breast MRI, and 5 of these patients (29.4%) had pathologically positive nodes. The sensitivity and specificity of breast MRI in the neoadjuvant endocrine therapy cohort were both 66.7% (Table 3). The positive and negative predictive values were 62.5 and 70.6%, respectively, and the overall accuracy was 66.7%. The sensitivity and NPV were significantly higher in this group compared to the neoadjuvant chemotherapy group, while overall accuracy was similar (Table 3).

Sensitivity and PPV were significantly higher in the clinically node positive patients compared to clinically node negative patients (87.5 vs. 42.9%, *p* = 0.0277 and 100 vs. 33.3%, *p* = 0.001, Table 3). NPV was significantly higher in the clinically node negative patients compared to clinically node positive patients (75 vs. 0%, *p* = 0.0002, Table 3). Overall accuracy in the clinically node negative cohort was 60%; accuracy in the clinically node positive cohort could not be calculated because there were no pathologically node negative patients in this group.

### Clinically node negative cases: burden of nodal disease and pathologic upgrade rates

Among the 49 clinically node negative patients, 35 (71.4%) had normal lymph nodes on post-treatment MRI. Of these patients, 23 (65.7%) were pathologically node negative, 9 (25.7%) had 1–3 positive nodes, and 3 (8.6%) had ≥4 positive nodes (Table 4). For the 14 clinically node negative patients with abnormal lymph nodes on MRI, 9 (64.3%) were node negative, 2 (14.3%) had 1–3 positive nodes, and 3 (21.4%) had ≥4 positive nodes. The appearance of lymph nodes on post-treatment MRI was not significantly associated with burden of nodal disease (Table 4).

**Table 4:** Association between nodal disease burden on pathology and MRI findings in clinically node negative and clinically node positive patients.

	0 Positive nodes	1–3 Positive nodes	≥4 Positive nodes	*P*-Value
Clinically node negative				0.38
Normal post-treatment breast MRI (*N* = 35)	23 (65.7%)^b^	9 (25.7%)	3 (8.6%)	
Abnormal post-treatment breast MRI (*N* = 14)^a^	9 (64.3%)	2 (14.3%)	3 (21.4%)	
Clinically node positive				0.034
Normal post-treatment breast MRI (*N* = 12)	1 (8.3%)	9 (75.0%)	2 (16.7%)	
Abnormal post-treatment breast MRI (*N* = 18)	2 (11.1%)	5 (27.8%)	11 (61.1%)	

^a^Abnormal post-treatment MRI defined as any abnormal finding on post-treatment MRI, including cortical thickening ≥3 mm, rounded morphology, loss of fatty hilum, and/or asymmetry.

^2^Values reported as *N* (%) unless otherwise stated.

**Fig. 3:**
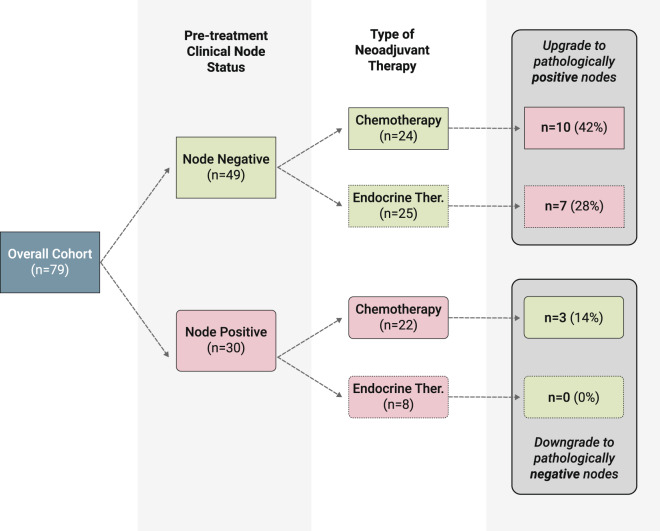
Upgrade and downgrade rates following neoadjuvant therapy by clinical node status. Pathologic node status following neoadjuvant chemotherapy or endocrine therapy in clinically node negative and positive patients.

We evaluated the upgrade rate to pathologically positive nodes in the clinically node negative cases in both the neoadjuvant chemotherapy and endocrine therapy cohorts (Fig. 3). A total of 49 patients (62.0%) were clinically node negative, with 24 (49.0%) receiving neoadjuvant chemotherapy and 25 (51.0%) receiving neoadjuvant endocrine therapy. Of the 24 patients who received neoadjuvant chemotherapy, 10 (41.7%) were found to have pathologically positive nodes at the time of surgery. Of the 25 patients who received neoadjuvant endocrine therapy, seven (28.0%) had positive nodes at the time of surgery. Taken together, a total of 17 cases upgraded to pathologically positive nodes, of whom 16 (94.0%) had the classic subtype of ILC and 1 (6.0%) had the pleomorphic subtype.

### Clinically node positive cases: burden of nodal disease and pathologic downgrade rates

Among the 30 clinically node positive patients, 12 (40.0%) had normal lymph nodes on post-treatment MRI. Of these patients, 1 (8.3%) was node negative, 9 (75.0%) had 1–3 positive nodes, and 2 (16.7%) had ≥4 positive nodes. There were no clinical or pathologic characteristics that predicted resolution of the imaging abnormality on MRI in these clinically node positive patients. Additionally, a total of 18 patients had abnormal lymph nodes on post-treatment MRI; 2 (11.1%) had pathologically negative nodes, 5 (27.8%) had 1–3 positive nodes, and 11 (61.1%) had ≥4 positive nodes. Abnormal lymph nodes on post-treatment MRI was significantly associated with a higher burden of nodal disease in these clinically node positive patients (*p* = 0.034).

Lastly, we evaluated the downgrade rate to pathologically negative nodes in the clinically node positive cases in both the neoadjuvant chemotherapy and endocrine therapy cohorts. Of the 30 patients who were clinically node positive, 22 (73.3%) were treated with neoadjuvant chemotherapy, and eight (26.7%) were treated with neoadjuvant endocrine therapy (Fig. 3). Of the 22 patients who received neoadjuvant chemotherapy, only three (13.6%) were found to have pathologically negative nodes (nodal complete response) at the time of surgery. The ILC subtypes in these patients were pleomorphic in 2 (66.7%) and classic in 1 (33.3%). In the endocrine therapy cohort, no patients were downgraded to pathologically negative nodes at the time of surgery.

## Discussion

In this unique cohort of patients with ILC, we evaluated the performance of breast MRI in predicting the status of axillary nodes in the setting of either neoadjuvant chemotherapy or neoadjuvant endocrine therapy and found that the overall accuracy ranged from 45.5–66.7%. Our results suggest that breast MRI had significantly higher sensitivity and NPV for axillary nodal status in patients receiving neoadjuvant endocrine therapy compared to neoadjuvant chemotherapy, although overall accuracy was similar. However, clinical nodal status impacts the pre-test probability of having positive or negative nodes after neoadjuvant therapy and therefore impacts test performance.

Among patients receiving neoadjuvant chemotherapy, MRI had significantly higher sensitivity and PPV in clinically node positive patients but significantly higher specificity and NPV in clinically node negative patients. These findings suggest that a negative post-treatment MRI is more likely to be accurate in a clinically node negative patient, whereas a positive post-treatment MRI is more likely to be accurate in a clinically node positive patient. Importantly, overall accuracy was low in both the clinically node negative and positive patients who received neoadjuvant chemotherapy (54.2 and 45.5%, respectively).

Similarly, MRI performed differently based on clinical node status in the cohort receiving neoadjuvant endocrine therapy. In this case, sensitivity and PPV were significantly higher in the clinically node positive patients, suggesting that any abnormal appearance of lymph nodes in clinically node positive patients after neoadjuvant endocrine therapy is almost always an indicator of residual disease, as the PPV was 100%. This is consistent with very low complete nodal response rates to neoadjuvant endocrine therapy in this population. Among the clinically node negative patients who received neoadjuvant endocrine therapy, NPV was 75%, suggesting that a negative MRI was correct in most of these patients.

While the accuracy for predicting lymph node status was low overall, we did find that nodal appearance on breast MRI may be used to identify patients with a high burden of nodal disease. The presence of ≥4 positive lymph nodes impacts systemic treatment decisions and is particularly important for patients with ILC tumors, which are often genomically low-risk but might benefit from chemotherapy based on clinical risk^7,19^. We found that only a small proportion of clinically node negative patients had ≥4 positive nodes after neoadjuvant therapy, regardless of the appearance of nodes on breast MRI. However, among the clinically node positive cohort, the appearance of nodes on post-treatment MRI reliably distinguished between those with fewer than 4 positive nodes versus ≥4 positive nodes. Thus, while our findings do not support the broad use of breast MRI for planning the approach to axillary surgery in ILC, there may be a role for its use in pre-operative treatment strategy in clinically positive patients with a high burden of nodal disease, as well as patients treated with endocrine therapy.

Investigators have reported the accuracy of nodal evaluation with breast MRI to range from 60 to 87% in patients with IDC after neoadjuvant chemotherapy^8–13^. We found that MRI is less accurate for patients with ILC, which is consistent with prior literature showing lower imaging sensitivity overall for this type of tumor^16^. The low accuracy in our chemotherapy-treated cohort differs from some prior reports that have shown breast MRI to have good performance in assessment of response after neoadjuvant chemotherapy. However, the literature reports higher MRI accuracy in patients with HER2 positive or triple-negative tumors, which are more commonly treated with chemotherapy^20^. Importantly, invasive lobular cancer is mostly ER positive/HER2 negative, likely accounting for this lower accuracy.

A striking finding in this study was the high upgrade rate among clinically node negative patients across both cohorts. In clinically node negative patients who are not treated with neoadjuvant therapy, the reported rate of nodal involvement at surgical excision ranges from 15 to 30% in studies largely consisting of cases of IDC^21,22^. In our patient population, we found that 41.7% of clinically node negative patients who received neoadjuvant chemotherapy had nodal disease at surgery despite thorough pre-treatment imaging evaluation with breast MRI and axillary ultrasound in a majority of patients. This finding suggests that nodal involvement is common and clinical detection of nodal disease is low in ILC patients who are deemed eligible for neoadjuvant chemotherapy. The upgrade rate was lower in the clinically node negative cohort who received neoadjuvant endocrine therapy, but still high at 28.0%. This finding is again consistent with negligible axillary response to neoadjuvant endocrine therapy. Both the high upgrade rate among clinically node negative patients and the low rate of nodal complete response in clinically node positive patients highlight to need for more effective neoadjuvant therapies for this unique cohort.

Our study has several strengths, including the availability of two unique cohorts of patients with ILC treated with neoadjuvant therapy and centralized radiology review of MRIs by two expert breast radiologists. However, there are important limitations to our study, including its retrospective nature. Determination of clinical nodal status depended on clinical decisions regarding level of suspicion and choice to obtain needle biopsy or not, which could result in underestimation of clinical stage. However, the high rate of pre-treatment breast MRI utilization in this cohort (89.9%) suggests thorough pre-treatment evaluations were generally conducted. Surgical procedure of SLNB versus axillary dissection was at the discretion of treating physicians and patients and could impact ascertainment rates of pathologically positive nodes. Additionally, measurements of cortical thickness in millimeters on MRI can be difficult given slice thickness, and as such we used cortical thickness as a binary variable instead of continuous. Finally, technical differences in the performance of breast MRI over the study period could impact accuracy, but small sample size prevented us from evaluating trends over time.

In summary, this study is one of the first to investigate the use of breast MRI in the evaluation of nodal response after neoadjuvant chemotherapy or endocrine therapy in patients with ILC. Our findings indicate that undetected residual nodal disease in patients with ILC treated with neoadjuvant therapy is high and the accuracy of breast MRI to evaluate nodal response is low, suggesting that axillary findings on post-treatment breast MRI should not be used to plan the surgical approach to the axilla. However, using MRI to identify patients with ILC and a high burden of nodal disease is more promising and could help guide systemic therapy decisions. Increased representation of patients with ILC in clinical trials and development of novel imaging tools is needed to improve outcomes and enhance care for this unique population.

## Methods

### Study cohort

We conducted a retrospective analysis of a cohort of women who received neoadjuvant therapy for ILC at the University of California, San Francisco (UCSF), between 2006 and 2019. The study was approved by the UCSF Institutional Review Board, and informed consent was waived because no patients were contacted directly for this study. We queried a prospectively maintained surgical database and identified stage 1–3 ILC patients who underwent either neoadjuvant chemotherapy or endocrine therapy and underwent post-treatment dynamic contrast enhanced breast MRI prior to surgery as part of routine clinical care. ILC was diagnosed by routine histology with selective E-cadherin staining. We excluded patients with de novo stage 4 disease and those with less than 6 months of follow-up time. At our institution, the routine evaluation of the axilla prior to neoadjuvant therapy includes a physical exam with selective axillary ultrasound and breast MRI. Patients were deemed clinically node positive if they had a positive axillary lymph node diagnosed through core biopsy or fine needle aspiration (FNA) prior to neoadjuvant treatment. After completion of neoadjuvant therapy, all patients underwent either sentinel lymph node biopsy (SLNB) or axillary lymph node dissection (ALND) at the discretion of the treating surgeon. Final nodal status was determined by surgical pathology with hematoxylin-eosin stains and selective cytokeratin staining. Among pathologically positive lymph nodes, the size of the largest tumor deposit and presence of extracapsular extension were recorded from surgical pathology reports.

### Breast MRI review

We restricted the time period of our analysis to 2006 and onward to account for technical differences in the performance of MRI, as unilateral breast MRI was more commonly performed than bilateral breast MRI prior to that year. All breast MRIs met the American College of Radiology’s (ACR) practice parameter guidelines^23^. Breast MRI was performed with the patient in prone position according to our institutional protocol on either a 1.5 or 3.0 Tesla (T) magnet (Signa Echospeed; General Electric Medical Systems, Milwaukee, WI and Avanto; Siemens Medical Solutions, Erlangen, Germany) using a dedicated 16 channel breast coil (MRI Devices, Waukesha, WI). Prior to 2019, Gadobutrol (Gadavist®; Bayer Schering Pharma, Berlin, Germany) and gadoterate meglumine (Magnevist; Schering, Berlin, Germany) were used as the intravenous contrast agents, while gadoterate meglumine (Gd-DOTA) [Magnescope® in Japan, Dotarem® in other countries] was used after 2019 at a dose of 0.1 mmol kg^–1^ of body weight, followed by a 20-ml saline flush.

Each breast MRI was reviewed by two fellowship-trained breast radiologists in the UCSF Department of Radiology, with 3–7 years of experience, blinded to the clinical and pathological nodal status. The following features were used to classify lymph nodes as abnormal: cortical thickening (≥3 mm), presence of rounded morphology, loss of normal fatty hilum, and asymmetry when compared to the contralateral axilla (Fig. 1a and b). Our primary predictor was the presence of any abnormal axillary nodes on breast MRI, as defined by the presence of at least one abnormal nodal feature. For all cases with at least one abnormal node, the number and anatomic level of abnormal nodes were recorded.

### Statistical analysis

Data were analyzed in Stata 14.2 (StataCorp LLC, College Station, TX, USA), using the Chi-squared test for categorical variables and analysis of variance for continuous variables. We used contingency tables to calculate the sensitivity, specificity, negative predictive value (NPV), positive predictive value (PPV), and overall accuracy of abnormal nodal features on breast MRI in predicting axillary lymph node status following neoadjuvant therapy. We grouped patients by type of neoadjuvant therapy (chemotherapy versus endocrine therapy) and clinical nodal status (clinically node positive versus clinically node negative) and used a two-sample test of proportions to compare diagnostic test performance by group.

### Reporting summary

Further information on research design is available in the Nature Research Reporting Summary linked to this article.

## Supplementary information

Reporting Summary Checklist

## Data Availability

The data that support the findings of this study, contain clinical outcomes for which IRB requires approval prior to analysis. Therefore, the data are not publicly available. The data will be made available to authorized researchers who have obtained institutional review board (IRB) approval from their own institution and from the University of California, San Francisco (UCSF) IRB. For data access requests, please contact the corresponding author, Dr. Rita Mukhtar, email address: rita.mukhtar@ucsf.edu. The data generated and analyzed during this study are described in the following metadata record: https://doi.org/10.6084/m9.figshare.13643087^24^.

## Code availability

All statistical analyses were performed using Stata 14.2 (StataCorp LLC, College Station, TX, USA). Code is available upon reasonable request from any qualified researcher.
